# Improved Assessment and Prediction of Groundwater Drinking Quality Integrating Game Theory and Machine Learning in the Nyangchu River Basin, Southwestern Qinghai–Tibet Plateau

**DOI:** 10.3390/toxics13110985

**Published:** 2025-11-16

**Authors:** Xun Huang, Xiyong Wu, Weiting Liu, Denghui Wei, Ying Wang, Hua Wu, Yangshuang Wang, Boyi Zhu, Qili Hu, Yunhui Zhang, Wei Wang

**Affiliations:** 1 Faculty of Geosciences and Engineering, Southwest Jiaotong University, Chengdu 611756, China; 2 Yibin Research Institute, Southwest Jiaotong University, Yibin 644000, China; 3 Sichuan Province Engineering Technology Research Center of Ecological Mitigation of Geohazards in Tibet Plateau Transportation Corridors, Chengdu 611756, China; 4 College of Engineering, Tibet University, Lhasa 850000, China; 5 School of Chemical and Environmental Engineering, Sichuan University of Science & Engineering, Zigong 643000, China; 6 The 1st Geological Brigade of Sichuan, Chengdu 610032, China

**Keywords:** hydrochemical analysis, game theory, machine learning, groundwater quality prediction

## Abstract

To address the limitations of traditional groundwater quality assessment and prediction methods, this study integrates game theory and machine learning to investigate the drinking quality of groundwater in the southwestern Qinghai–Tibet Plateau. The results showed that the groundwater in the study area is generally weakly alkaline (mean pH: 8.08) and dominated by freshwater (mean TDS: 302.58 mg/L), with hardness levels mostly ranging from soft to medium. Major cations follow the concentration order: Ca^2+^ > Na^+^ > Mg^2+^ > K^+^; anions are in the sequence of HCO_3_^−^ > SO_4_^2−^ > Cl^−^. The hydrochemical type is mainly Ca-HCO_3_. A few samples exceed the limit values specified in the Groundwater Quality Standard. Through multivariate statistical analysis, ion ratio analysis, and saturation index calculations, water-rock interaction is identified as the primary factor influencing groundwater chemistry. It consists of carbonate dissolution and silicate weathering, accompanied by cation exchange. The water quality index improved based on game theory, integrated subjective weights (from analytic hierarchy process) and objective weights (from entropy-weighted method), shows that the overall groundwater quality in the study area is good: 95.97% of the samples are high-quality water (WQI ≤ 50), more than 99% of the samples have a WQI < 150, which is suitable as drinking water sources; only 0.81% of the samples are of extremely poor quality, presumably related to local pollution. Linear regression achieved the best performance (R^2^ = 0.99, RMSE≈0.00) with strong stability, followed by support vector machines (test R^2^ = 0.98), while the extreme gradient boosting model showed overfitting. This study provides a scientific basis for groundwater management in river basins.

## 1. Introduction

Groundwater is one of the most important sources of drinking water worldwide, characterized by its relative stability and cleanliness [1,2]. Groundwater quality assessment is a core link in water resources management, environmental protection, and sustainable social development. It plays a crucial role in safeguarding human health and public health security, supporting the sustainable development of industrial and agricultural production, and maintaining the hydrological cycle and biodiversity of ecosystems [3,4]. In addition, groundwater quality prediction utilizes scientific methods to forecast the evolution of groundwater quality and the degree of pollution risk over a specified future period, providing forward-looking guidance for groundwater management [5,6].

The primary assessment methods for groundwater quality can be divided into the single-factor method and the comprehensive index method. The single-factor method focuses on comparing the concentration of a single pollutant in water with the assessment standards [7]. It determines the overall water quality based on the principle of “grading by the worst-performing factor”. Its advantages lie in simple logic, convenient calculation, and intuitive results, which enable the quick identification of key pollution factors in water. However, it overlooks the synergistic effects between pollutants, resulting in conservative assessment results that fail to accurately reflect the comprehensive pollution level of water [8,9]. In contrast, the comprehensive index method integrates information from multiple pollutant indicators by assigning weights, effectively mitigating this defect and rendering the results more scientific, thereby being widely adopted [10,11,12]. Nevertheless, the results of the index method depend on the weights of indicators. Traditional index methods determine weights based on expert knowledge and experience, which are highly subjective [13,14,15,16,17]. To address this, the entropy-weight water quality index performs objective weighting of indicators based on the theory of information entropy, avoiding artificial subjective biases and making the assessment results more consistent with reality [18,19,20]. However, abnormal data values may significantly affect indicator weights in this method, and if there is a high correlation between indicators, duplicate weighting may occur. In response to this, the concept of game theory can be applied to coordinate subjective and objective indicator weights and achieve a balance in water quality assessment [21,22]. In addition, the advancement of technology has enabled the integration of traditional assessment methods with big data, artificial intelligence, and other technologies, resulting in the development of more efficient and accurate assessment systems [23,24,25].

Groundwater quality prediction methods can be categorized into three types: empirical statistical methods, numerical simulation methods, and machine learning methods [26,27]. The empirical statistical methods establish regression models for prediction based on the statistical relationship between historical water quality monitoring data and influencing factors. They are characterized by being data-driven and easy to operate. However, they fail to reflect the physical migration and chemical transformation processes of pollutants in groundwater, resulting in low accuracy under complex hydrogeological conditions [28]. The numerical simulation methods, based on the principles of groundwater dynamics and hydrochemistry, construct mathematical models that reflect the “groundwater flow field-pollutant migration and transformation”. They simulate the future evolution of water quality through numerical calculations and are currently the most mainstream and accurate prediction methods [29,30,31]. Nevertheless, they have high requirements for hydrogeological data, involve complex parameter calibration, and incur long modeling cycles and high costs. In contrast, machine learning methods extract nonlinear correlations from monitoring data using algorithms, eliminating the need to elucidate physical and chemical mechanisms [32,33,34]. While ensuring a certain level of accuracy, they also offer the advantages of cost-effectiveness and efficiency.

The study area is situated on the Qinghai–Tibet Plateau, where groundwater serves as a vital source of drinking water for local residents. Its hydrochemical characteristics, quality status, and predictive modeling remain poorly understood. Moreover, the quantity of groundwater samples is limited and unevenly distributed due to the limitations of objective conditions. The current assessment methods are likely to underestimate or overestimate the weight of a certain indicator, thereby leading to incorrect conclusions. Therefore, this study collected groundwater samples from the study area, aiming to: (1) investigate groundwater formation mechanisms, (2) evaluate drinking water quality using an improved index, and (3) predict water quality through machine learning. Its novelty lies in using the concept of game theory to balance the weights of indicators and thereby evaluate water quality, reducing the deviations caused by the small sample size and poor quality of the data. And through machine learning algorithms, a water quality prediction model for the study area was established under the condition of lacking objective data. The findings will contribute to the scientific management of local groundwater resources.

## 2. Materials and Methods

### 2.1. Study Area

**Figure 1:**
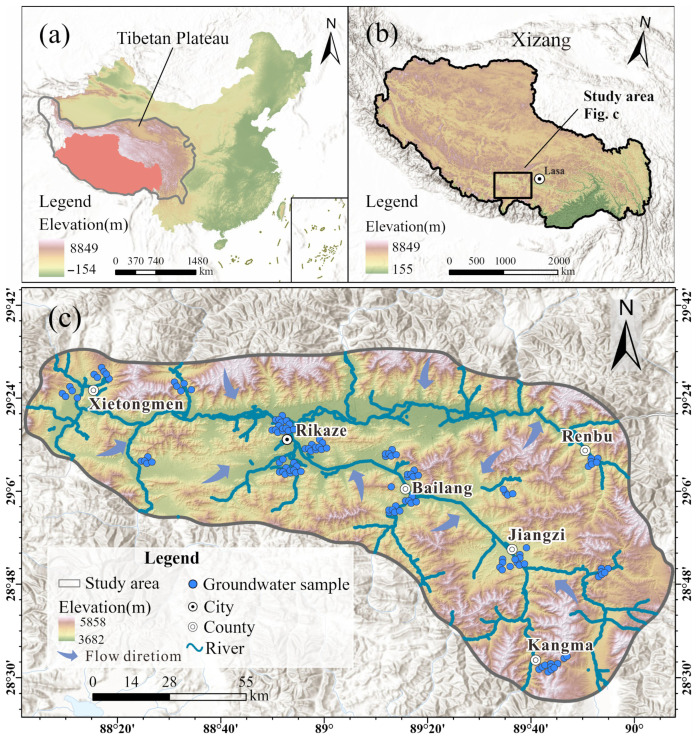
(**a**) Location of the Qinghai–Tibet Plateau within China, (**b**) Location of the study area within the Qinghai–Tibet Plateau; (**c**) The distribution of groundwater samples within the study area.

The study area is located in southwestern Tibet, between 27°13′ N and 31°49′ N, and 80°01′ E and 90°20′ E (Figure 1). Characterized by a cool and cold climate, it falls under the plateau temperate semi-arid climate zone. The annual average temperature ranges from approximately 0 °C to 6.5 °C, with an annual average precipitation of about 421.9 mm, primarily occurring during the rainy season (May to September). The area is rich in water resources, including major rivers such as the Yarlung Zangbo River (the largest river in Tibet) and the Nyangchu River. Most of the lakes here are inland lakes, mainly saltwater lakes or salt lakes, whose water is rich in mineral components such as salt, boron, calcium, and sodium. The types of land resources are diverse, including cultivated land, grasslands, woodlands, wastelands, lakes, and swamps. Cultivated land is mainly concentrated in the valley areas along the banks of the Yarlung Zangbo River and the Nyangchu River. Local human activities are primarily associated with agriculture and animal husbandry, including crop cultivation and livestock grazing. These practices may influence groundwater quality through fertilizer application, irrigation return flow, and surface runoff. Industrial activities are relatively limited and mainly concentrated around the urban area, where small-scale mining, food processing, and construction material industries are present. Moreover, domestic wastewater discharge from residential settlements may also alter the hydrochemical characteristics of the groundwater.

The study area is situated between the middle sections of the Himalayan Mountain Range and the Gangdise-Nyainqêntanglha Mountain Range. With relatively high terrain in the north and south, its topography is complex and diverse, consisting essentially of high mountains, wide valleys, and lake basins, with an average altitude of over 4000 m. The geological characteristics are complex and varied, and their formation is closely related to the uplift of the Qinghai–Tibet Plateau and the tectonic movement of the Himalayan Mountains. Overall, it exhibits obvious signs of intense tectonic activity and significant north–south zonation differences. The southern part is dominated by ancient metamorphic rock series and weakly metamorphosed sedimentary rock series, which mainly include gneiss, marble, shale, slate, and phyllite. The central part is mainly composed of a mixed distribution of intermediate-acid intrusive rocks and sedimentary rocks from the late Mesozoic to the early Cenozoic, including granite, sandstone, and conglomerate. Cenozoic continental sedimentary rocks, including conglomerate, sandstone, siltstone, and mudstone, dominate the northern part. Quaternary unconsolidated sediments are also distributed in the valley areas. In addition, the primary recharge sources of groundwater in the study area include precipitation, glacial meltwater, and river recharge, while the discharge pathways comprise springs, wells, and rivers, among others.

### 2.2. Groundwater Sampling and Experimental Testing

A total of 129 groundwater samples were systematically collected in May 2024 with the well depths ranging 5–20 m to ensure representative coverage of the study area. The geographic coordinates of each sampling site were accurately determined and recorded using a handheld Global Positioning System (GPS) device. The spatial distribution of all sampling locations is illustrated in Figure 1. Prior to sampling, groundwater was pumped for more than 30 min to eliminate the influence of stagnant water. Field measurements of physicochemical parameters, including pH and total dissolved solids (TDS), were performed using a portable multi-meter (Multi 3400i, WTW, Munich, Germany). The 500 mL polyethylene bottles used for sample collection were first thoroughly rinsed with distilled water and subsequently rinsed more than three times with groundwater from the sampling point to ensure the representativeness and reliability of the collected samples. During sampling, bottles should be filled with groundwater to prevent air entrapment and minimize potential interference. Moreover, to evaluate sampling and analytical precision, field duplicate (co-located) samples and blanks at 10% of the sites were also collected. The blanks included field/equipment rinse blanks to check for contamination during sampling and handling. The collected bottles were stored at 4 °C and quickly transported (within a week) to the Sichuan Geological Survey Institute for further hydrochemical examination.

Cation concentrations (K^+^, Na^+^, Ca^2+^, Mg^2+^) were analyzed using inductively coupled plasma mass spectrometry (ICPMS7500ce, Agilent, Shanghai, China), whereas anion concentrations (Cl^−^, SO_4_^2−^, NO_3_^−^, F^−^) were determined via ion chromatography (LC-10Advp, Shimadzu, Jinan, China). HCO_3_^−^ levels and total hardness (TH) were quantified by titration. For ion chromatography/ICP-MS determinations, two independent runs per sample were performed. If the relative percent difference (RPD) between runs exceeded 10% for major ions/parameters or 15% for trace elements, the sample was re-analyzed and the mean of the acceptable runs was reported. Moreover, to verify the reliability of the groundwater measurements, an anion–cation charge balance calculation was performed for all water samples using Equation (1), and the charge balance error was maintained within ±5%.

(1) $$\mathrm{IBE}\left(\%\right)=\frac{\sum \mathrm{cations}-\sum \mathrm{anions}}{\sum \mathrm{cations}+\sum \mathrm{anions}}\times 100$$

### 2.3. Improved Drinking Water Quality Evaluation Approach Based on Game Theory

Game theory, an important modern mathematical optimization strategy, is utilized to calculate the proportion between the subjective weights (*W*_1_) derived from the analytic hierarchy process and the objective weights (*W*_2_) calculated by the entropy-weighted method, thus computing the comprehensive parameter weights and evaluating the groundwater quality accordingly [35,36,37]. The calculation process is given in Equations (2)–(6).

(2) W1W1TW1W2TW2W1TW2W2Td1d2=W1W1TW2W2T

(3) $$\left\{\begin{array}{c} d_{1}^{*}=\frac{d_{1}}{d_{1}+d_{2}}\\ d_{2}^{*}=\frac{d_{2}}{d_{1}+d_{2}} \end{array}\right.$$

(4) $$W=W_{1}d_{1}^{*}+W_{2}d_{2}^{*}$$

(5) $$\left\{\begin{array}{c} q_{i}=\frac{C_{i}}{S_{i}}\times 100\\ q_{\mathrm{pH}}=\left\{\begin{array}{c} \frac{C_{\mathrm{pH}}-7}{\max\{C_{\mathrm{pH}}-7\}}\times 100,\;pH>7\\ \frac{7-C_{\mathrm{pH}}}{\max\{{7-C}_{\mathrm{pH}}\}}\times 100,\;pH<7 \end{array}\right. \end{array}\right.$$

(6) $$\mathrm{WQI}=\sum_{i=1}^{m}\left(w_{i}\times q_{i}\right)$$

where the *d*_1_* and *d*_1_* represent the weight coefficient for subjective weights (*W*_1_) and objective weights (*W*_2_), respectively; *W* is the comprehensive parameter weights. The *q_i_*, *C_j_*, and *S_i_* denote the quantitative grading scale, concentration, and the corresponding hydrochemical parameter limit values specified in the Standard for Groundwater Quality (Class III) (GB/T 14848-2017) [38], respectively.

#### 2.3.1. Analytic Hierarchy Process

The subjective hydrochemical parameter weights are determined via the analytic hierarchy process (AHP), a multi-index subjective weighting method [39,40]. In this study, the target layer is the drinking water quality assessment. The criterion layer is divided into low-hazard indicators and medium-hazard indicators based on the hazard level of the indicators. In the solution layer, the low-hazard indicators include pH, TH, TDS, and seven major ions. These indicators pose minor hazards to human health and only cause harm when ingested at extremely high concentrations. The medium-hazard indicators include NO_3_^−^ and F^−^, which can cause significant harm when their concentrations reach a certain level. The construction of the judgment matrix is carried out through the scaling method, and the parameter weights in each matrix can be calculated as Equations (7) and (8). Additionally, each matrix must pass the consistency test as specified in Equation (9) (*CR* < 0.1).

(7) $$\bar{w_{i}}=\sqrt[{m}]{\prod_{j=1}^{m}a_{ij}}$$

(8) $$w_{i}=\frac{\bar{w_{i}}}{\sum_{j=1}^{m}w_{j}}$$

(9) $$\left\{\begin{array}{c} CI=\frac{\lambda_{max}-n}{n-1}\\ CR=\frac{CI}{RI} \end{array}\right.$$

where $\bar{w_{i}}$ represents the m-th power of the product of each row and m is the dimension of matrix; *w_i_* is the weight of each parameter. *CI* stands for consistency index, *λ_max_* and n are the maximum eigenvalue of the matrix and the number of parameters, respectively. In addition, *CR* is the consistency ratio, and *RI* can be obtained based on the value of n (Table 1).

#### 2.3.2. Entropy–Weighted Method

The objective parameter weights are calculated by the entropy weight method, and its specific process is as follows [41,42]: (1) Construct the parameter matrix X (Equation (10)); (2) Standardize matrix X according to Equation (11); (3) Calculate the information entropy (*e_j_*) and weight (*w_j_*) of each parameter (Equations (12)–(14)). Where the *x_ij_* represents the measured value of the jth parameter of the *i*th sample. The *m* and *n* are the number of samples and parameters. The addition of 0.00001 is to avoid *y_ij_* being 0.

(10) $$X=\left[\begin{array}{cccc} x_{11} & x_{12} & \ldots & x_{1n}\\ x_{21} & x_{22} & \ldots & x_{2n}\\ \vdots & \vdots & \ddots & \vdots \\ x_{m1} & x_{m2} & \ldots & x_{\mathrm{mn}} \end{array}\right]$$

(11) $$y_{ij}=\frac{x_{ij}-min\{x_{1j},x_{2j},\ldots ,x_{mj}\}}{max\{x_{1j},x_{2j},\ldots ,x_{mj}\}-min\{x_{1j},x_{2j},\ldots ,x_{mj}\}}+0.00001$$

(12) $$P_{\mathrm{ij}}=\frac{y_{\mathrm{ij}}}{\sum_{i}^{m}\;y_{\mathrm{ij}}}$$

(13) $$e_{j}=-\frac{1}{\ln m}\sum_{i=1}^{m}\left(P_{\mathrm{ij}}\times \ln P_{\mathrm{ij}}\right)$$

(14) $$w_{j}=\frac{1-e_{j}}{\sum_{i=1}^{n}\left(1-e_{j}\right)}$$

### 2.4. Machine Learning Algorithms for Water Quality Prediction

This study employed linear regression (LR), support vector machines (SVM), and extreme gradient boosting (XGB) to evaluate model performance and identify the optimal approach for WQI-based water quality prediction in the study area (Text S1). LR offers high computational efficiency and strong interpretability, but its prediction accuracy is limited and it may not adequately capture complex nonlinear relationships among hydrochemical parameters [43]. The SVM exhibits robust resistance to overfitting and are relatively insensitive to missing values. However, it is slower when training on large-scale datasets, less interpretable, and its predictive performance can be sensitive to kernel selection and parameter tuning [44]. XGB can effectively capture nonlinear relationships between water quality indicators and environmental factors with high accuracy, although its interpretability is lower than that of LR, and it may exhibit overfitting when dealing with limited or heterogeneous samples [45].

To comprehensively evaluate model accuracy, five performance indicators, Coefficient of Determination (R^2^), Root Mean Square Error (RMSE), Mean Absolute Error (MAE), Percentage of Non-Improved Absolute Scores (PNIAS), and Willmott Index (WI), were employed to assess the results. The indicators calculation methods are presented in Equations (15)–(17). R^2^ indicates the degree of fit and the consistency between predicted and observed values, ranging from 0 to 1, with values closer to 1 representing better agreement. RMSE and MAE quantify prediction errors, where smaller values correspond to higher prediction accuracy.

The dataset was split into a training set and a test set, comprising 82% and 18% of the data, respectively [46]. The test set was used to assess the model’s generalization performance. And the dataset’s input variables include pH, TDS, TH, K^+^, Na^+^, Ca^2+^, Mg^2+^, Cl^−^, SO_4_^2−^, HCO_3_^−^, NO_3_^−^, F^−^, and the calculated WQI, representing key characteristics of groundwater that influence water quality [47,48]. More principal introductions of the three models can be found in the Supplementary Materials.

In addition, to gain a deeper understanding of the model’s behavior and to identify the key water quality parameters, this study employed sensitivity analysis approach, One-at-a-Time analysis (OAT), to assess the influence of input variables on the prediction results. Detailed information on the model framework and the sensitivity analysis method can be found in the Supplementary Materials (Text S2).

(15) $$R^{2}=1-\frac{\sum_{i=1}^{n}\left({\mathrm{WQI}}_{\mathrm{predicted}}-{\mathrm{WQI}}_{\mathrm{calculated}}\right)}{\sum_{i=1}^{n}\left({\mathrm{WQI}}_{\mathrm{predicted}}-\bar{{\mathrm{WQI}}_{\mathrm{calculated}}}\right)}$$

(16) $$\mathrm{RMSE}=\sqrt{\frac{1}{n}\sum_{i=1}^{n}{\left({\mathrm{WQI}}_{\mathrm{predicted}}-{\mathrm{WQI}}_{\mathrm{calculated}}\right)}^{2}}$$

(17) $$\mathrm{MAE}=\frac{1}{n}\sum_{i=1}^{n}\left|{\mathrm{WQI}}_{\mathrm{predicted}}-{\mathrm{WQI}}_{\mathrm{calculated}}\right|$$

where WQI_predicted_ and WQI_calculated_ represent the ML-based predicted values and the calculated values of the WQI, respectively, and *n* is the number of samples.

## 3. Results

### 3.1. Hydrochemical Characteristics of Groundwater

**Figure 2:**
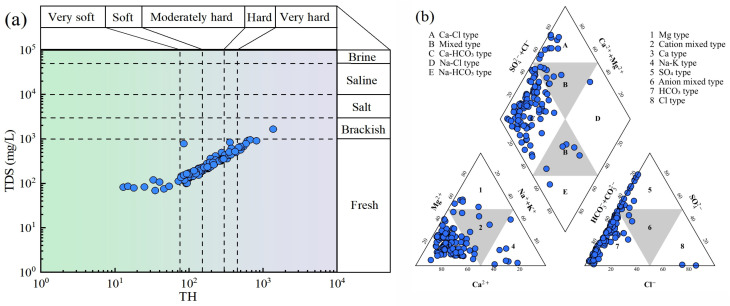
(**a**) Scatter diagram of TDS vs. TH; (**b**) Piper trilinear diagram of the groundwater.

The statistical characteristics of hydrochemical parameters are listed in Table 2. The pH value ranges from 7.00 to 9.10, with an average of 8.08. It suggests that the groundwater is slightly alkaline. A total of 12.90% samples exceeded the overall limit range of 6.5–8.5, which is possibly due to the mineral dissolution, human activities, and other factors. The coefficient of variation (CV) is 0.05, indicating a relatively stable pH level in the groundwater of the study area. The total dissolved solids (TDS) varied from 69.00 mg/L to 1660.00 mg/L, with 16.13% of samples exceeding the drinking water standard. Except for a few samples, all samples are from fresh water (Figure 2a). Furthermore, the total hardness (TH) value ranges from 15.00 mg/L to 1370.00 mg/L, with a mean value of 218.91. A total of 37.10% samples belong to soft water and moderate hard water, respectively. The CV (0.82) indicates high variability, reflecting significant differences in the contribution of rock weathering to calcium and magnesium in different regions, as well as the uneven distribution of total hardness.

Additionally, the contents of ions are as below: 0.15–20.30 (K^+^), 2.24–172.90 (Na^+^), 5.63–330.75 (Ca^2+^), 0.20–144.13 (Mg^2+^), 1.01–443.70 (Cl^−^), 1.45–951.66 (SO_4_^2−^), 39.90–519.75 (HCO_3_^−^), 0.02–73.50 (NO_3_^−^), 0.03–0.79 (F^−^). The average concentrations of major cations in groundwater are ranked as follows: Ca^2+^ (60.19 mg/L) > Na^+^ (17.08 mg/L) > Mg^2+^ (15.68 mg/L) > K^+^ (1.52 mg/L), while the major anions are HCO_3_^−^ (170.43 mg/L) > SO_4_^2−^ (82.29 mg/L) > Cl^−^ (10.46 mg/L). It noted that the concentrations of both major cations and anions showed high variability (CV > 0.36), and only a few samples exceeded the drinking water limit. Therefore, the groundwater in these areas may have been affected by either local excessive water–rock interaction or human pollution. Correspondingly, the sample points mainly fall within the areas indicating Ca and HCO_3_^−^ in the Piper diagram, supporting the dominant hydrochemical type of Ca-HCO_3_ type (Figure 2b). The NO_3_^−^ and F^−^ in groundwater are usually related to human activities, and their concentration ranges are 0.02–73.50 mg/L and 0.03–0.79 mg/L, respectively. Their CV values are higher than 0.5, and 4.03% of samples showed levels of NO_3_^−^ that exceeded the drinking water standard, implying potential pollution.

### 3.2. Improved Assessment of Groundwater Drinking Quality

**Figure 3:**
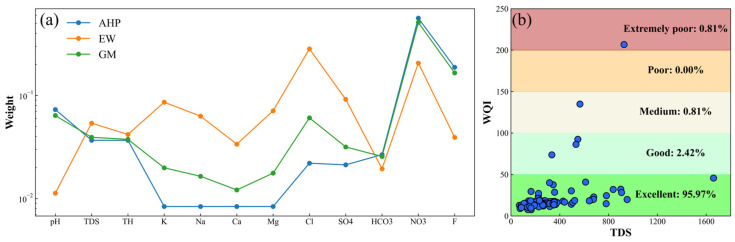
(**a**) Weight of hydrochemical indicators by different methods; (**b**) Results of drinking water quality assessment.

Subjective and objective parameter weights were calculated using the analytic hierarchy process (AHP) and the entropy-weighted method (EW), respectively. For AHP, judgment matrices were constructed separately for the criterion layer and the solution layer (Tables S1–S3), and the Consistency Ratio (CR) values of all matrices were less than 0.1 (0, 0.08, and 0), meeting the consistency requirement. Subsequently, improved parameter weights were obtained based on game theory (GM) and the hydrochemical parameter weights derived from the two aforementioned methods (Figure 3a). The weight of GM is generally located between that of AHP and EW, effectively avoiding the bias of a single method towards the water chemical indicators.

The groundwater drinking quality index (WQI) was calculated using the improved parameter weights. As shown in Figure 3b, the WQI varied from 7.52 to 206.74, with an average value of 20.71. More than 95% of samples are considered to have excellent water quality (WQI ≤ 50), and the WQI of over 99% of samples is below 150, suggesting that the groundwater quality within the study area is generally good and suitable for use as a drinking water source. In addition, only one sample reaches an extremely poor rating, which may be related to local pollution. In general, the groundwater quality in the study area is good, but there is human-induced pollution in some areas. It should not be used as drinking water.

## 4. Discussion

### 4.1. Hydrochemical Process and Controlling Factors

#### 4.1.1. Multivariate Statistical Analysis

Correlation and principal component analysis are widely employed to explore the potential connections and common sources among different ion components, thereby analyzing the hydrochemical processes of groundwater [49,50]. The Pearson coefficient is used to measure the correlation between variables, with its range spanning from −1 to 1. The two ends represent a completely positive correlation and a negative correlation, respectively. Additionally, the sample data passed the Kaiser-Meyer-Olkin (KMO = 0.51 > 0.50) test and Bartlett’s test of sphericity (*p* < 0.05), suggesting that factor analysis can be conducted.

**Figure 4:**
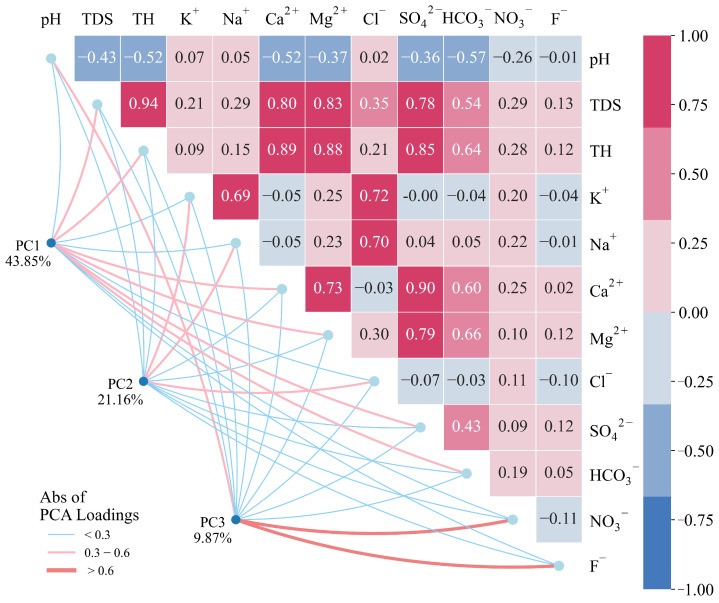
PCA-CA diagram of hydrochemical indicators in the groundwater.

As shown in Figure 4, there is a significant positive correlation between Cl^−^ and Na^+^ (0.70), which is most likely attributed to the dissolution of evaporite minerals (e.g., halite). In contrast, the correlation between K^+^ and Na^+^ may result from the weathering of silicate minerals. K^+^ and Na^+^ are released during the weathering process of these minerals. For instance, potassium feldspar releases K^+^ upon weathering, while albite releases Na^+^. When these minerals occur in the same geological environment under similar weathering conditions, the aforementioned ions exhibit a correlation in groundwater. Besides this mechanism, the correlation between K^+^ and Na^+^ may also indicate cation exchange reactions between groundwater and surrounding rocks. Furthermore, the correlations among Cl^−^, Na^+^, and K^+^ might imply the influence of anthropogenic factors; for example, domestic sewage discharge, industrial wastewater, and leachate from landfills may also contain these ions. In addition, positive correlations are observed among HCO_3_^−^ and Ca^2+^, Mg^2+^ (0.60–0.73), which are characteristic of the weathering and dissolution of carbonate rocks (e.g., calcite (CaCO_3_) and dolomite (CaMg(CO_3_)_2_). The correlation between Ca^2+^ and SO_4_^2−^ usually indicates the dissolution of gypsum (CaSO_4_·2H_2_O) or anhydrite (CaSO_4_). In addition, three principal components (PC1–PC3) with eigenvalues greater than 1 were extracted, and their variance explanations rates were 43.85%, 21.16%, and 9.87%, respectively. Where the PC1 is related to Ca^2+^, Mg^2+^, HCO_3_^−^, and SO_4_^2−^, which may represent the source of carbonate weathering and dissolution; PC2, associated with Na^+^, K^+^, and Cl^−^, is considered as the source of silicate dissolution; PC3 is characterized by NO_3_^−^ and F^−^, which might be related to human activities.

#### 4.1.2. Ion Source Analysis

The analysis of ion ratios is a core tool for identifying the formation process of the main ion components in groundwater, as the ion compositions formed by different interaction processes exhibit inherent differences [51,52]. It can reveal the interaction between groundwater and rocks, the degree of evaporation and concentration, and whether it is affected by human activities by calculating the concentration ratios of the main ions in groundwater.

**Figure 5:**
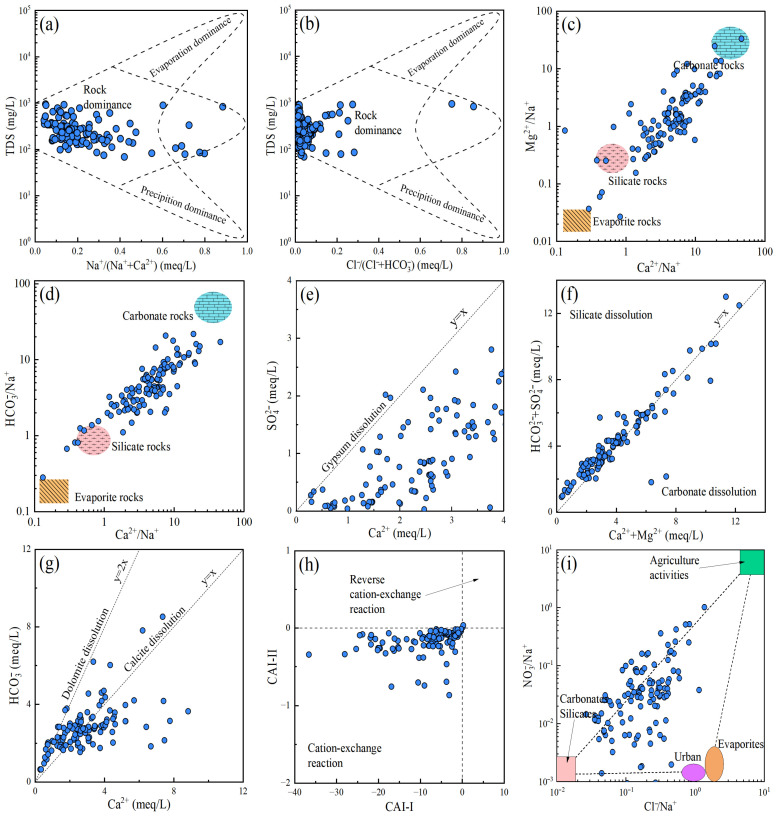
Ion ratio diagrams of hydrochemical components. (**a**,**b**) Gibbs diagrams; (**c**) (Mg^2+^/Na^+^) vs. (Ca^2+^/Na^+^); (**d**) (HCO_3_^−^/Na^+^) vs. (Ca^2+^/Na^+^); (**e**) SO_4_^2−^ vs. Ca^2+^; (**f**) (Ca^2+^+Mg^2+^) vs. (HCO_3_^−^/SO_4_^2−^); (**g**) HCO_3_^−^ vs. Ca^2+^; (**h**) CAI-II vs. CAI-I; (**i**) (NO_3_^−^/Na^+^) vs. (Cl^−^/Na^+^).

The Gibbs diagram can effectively identify the main controlling factors of groundwater chemical components. As shown in Figure 5a,b, most of the sample points fall within the “Rock dominance” area, indicating that the groundwater chemical evolution in the study area is primarily controlled by the weathering of rocks [53,54]. Meanwhile, a few points have shifted to the right, suggesting that they are also influenced by both the evaporation concentration process and precipitation discharge. In addition, the Gaillardet end-member diagram serves to distinguish the dissolution effects of different rocks [55]. The sample points are mainly distributed in the “Carbonate rocks” and “Silicate rocks” areas, while a few points are in the “Evaporite rocks” area (Figure 5c,d). This indicates that the groundwater chemistry in the study area is primarily influenced by the weathering of carbonate and silicate rocks, with a negligible contribution from the weathering of evaporite rocks. This viewpoint can also be reflected via the ratio of SO_4_^2−^ and Ca^2+^. Only a few sample points are close to the line *y* = *x* (Figure 5e), suggesting that the SO_4_^2−^ and Ca^2+^ in the groundwater are only slightly affected by the dissolution of evaporite mineral gypsum [56,57]. At the same time, most of the sample points deviated, indicating that other sources, such as the oxidation of sulfate minerals and the dissolution of other calcium-containing minerals, also affect the concentration of SO_4_^2−^ and Ca^2+^. In addition, the ratios of (HCO_3_^−^ + SO_4_^2−^) and (Ca^2+^ + Mg^2+^) for almost all the sample points are close to the line 1:1 (Figure 5f), indicating that the hydrochemical composition of groundwater is affected by the dissolution of both silicate and carbonate. Furthermore, the relationship between HCO_3_^−^ and Ca^2+^ can be utilized to identify the dissolution of carbonate minerals [58,59]. As depicted in Figure 5g, a portion of the samples falls between the lines *y* = *x* and *y* = 2*x*, suggesting that they are controlled by the dissolution of both dolomite and calcite.

In addition, some samples fall below *y* = *x*, indicating the influence of other sources on Ca^2+^ and HCO_3_^−^. The chloro-alkaline index (CAI) is employed to indicate the cation exchange and adsorption between groundwater and the surrounding rocks (Equation (18)). The CAI-I and CAI-II are lower than zero, indicating the occurrence of the cation exchange process, while the rest are positive values, which support the reverse reaction [60,61]. As shown in Figure 5h, most of the samples exhibit negative CAI-I and CAI-II, suggesting that the exchange between Ca^2+^ and Mg^2+^ in the groundwater and Na^+^ in the surrounding rocks. Additionally, the ratio of NO_3_^−^/Na^+^ to Cl^−^/Na^+^ at the sample points mainly falls between “carbonates silicates” and “agricultural activities” (Figure 5i), implying that the NO_3_^−^ within the groundwater primarily comes from agricultural practice and the dissolution of carbonates and silicates.

(Ca^2+^/Mg^2+^)_groundwater_ + 2(Na^+^)_rocks_ ⇋ 2(Na^+^)_groundwater_ + (Ca^2+^/Mg^2+^)_rocks_ (18)

**Figure 6:**
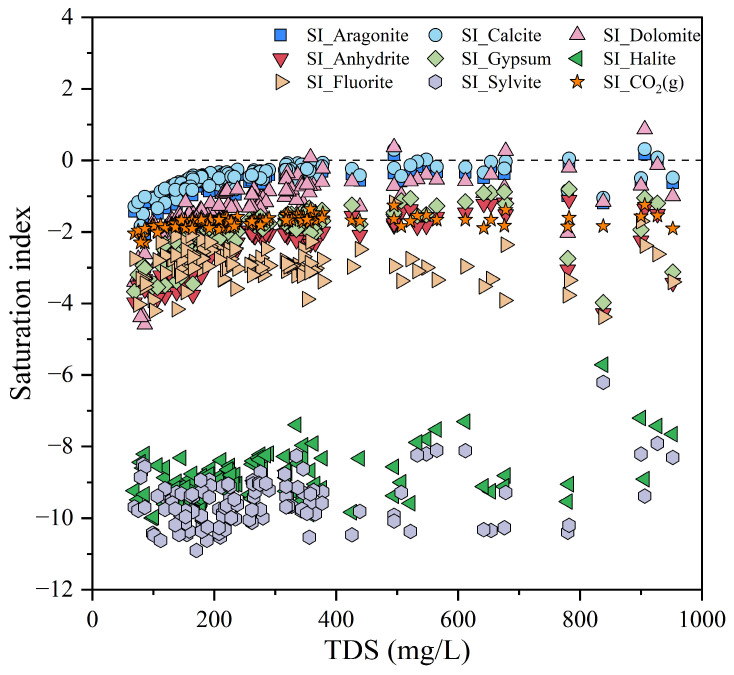
Saturation index of different minerals within the groundwater.

The saturation index (SI) serves as a key indicator for identifying the mineral dissolution equilibrium in groundwater, which can be calculated through the Phreeqc 3.0 software [62,63]. As shown in Figure 6, the saturation indices of most of the carbonate minerals (calcite, dolomite, and aragonite) in the samples are close to or slightly greater than 0, indicating that groundwater is close to saturating or in a weakly supersaturated state for these minerals. There may be a situation involving precipitation or dissolution, such as precipitation equilibrium of carbonate minerals. In addition, sulfate minerals such as Anhydrite and gypsum, as well as evaporite minerals such as sylvite and halite, have a SI mostly negative and with large absolute values, suggesting that the solution is not saturated with respect to these minerals. These minerals have a tendency to dissolve in water and are important sources of relevant ions in groundwater, such as SO_4_^2−^, Na^+^, and K^+^. The dissolved CO_2_(g) has not reached saturation either, implying the possibility that the CO_2_ in the atmosphere or in the soil may dissolve into the groundwater. The dissolution of CO_2_ will affect the acidity of the groundwater, thereby influencing the dissolution-precipitation equilibrium of carbonate and other minerals.

### 4.2. Water Quality Prediction Based on Machine Learning Approaches

To support effective water quality management in the study area, predictive models were developed using three machine learning algorithms, including LR, SVM, and XGB, based on the WQI. To ensure model robustness and mitigate overfitting, SVM and XGB underwent iterative parameter optimization, while LR was applied with its default settings. The final selected parameters for all models were presented in Table 3.

**Figure 7:**
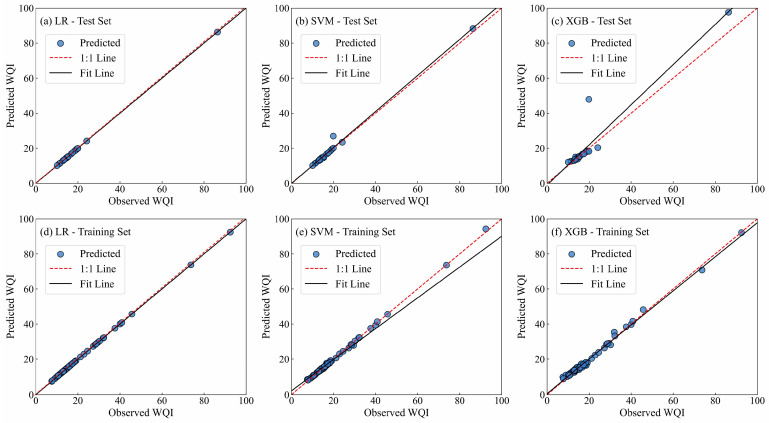
The relationship between calculated WQIs and predicted WQIs. (**a**) LR for test set; (**b**) SVM for test set; (**c**) XGB for test set; (**d**) LR for training set; (**e**) SVM for training set; (**f**) XGB for training set.

Figure 7 presented the results of the training and test sets for the three water quality prediction models, while the corresponding evaluation metrics for each model were summarized in Table 4. The LR model achieved outstanding predictive accuracy, as evidenced by R^2^ and WI values of 0.9999 for both the training and test datasets. Furthermore, the error metrics, including RMSE, MAE, and PBIAS (%), were all close to zero, indicating negligible deviation between the predicted and observed values. Due to the very high fitting performance of the LR model on both the training and test data, a 5-fold cross-validation (CV) procedure was applied to examine the model’s stability [64]. The results demonstrated that the evaluation metrics of LR on the training set were almost identical to those from 5-fold cross-validation, with ΔR^2^, ΔRMSE, and ΔMAE values approaching zero. This consistency highlighted the robustness and reliability of the model, supporting its applicability for predictive analysis.

In addition, the SVM model exhibited strong predictive performance, with R^2^ values of 0.98 and 0.97 for the test and training sets, respectively, and corresponding RMAE values of 1.55 and 4.01, as well as MAE values of 0.56 and 0.63. Five-fold cross-validation further confirmed the absence of overfitting, with performance differences between the training and validation sets (ΔR^2^, ΔRMSE, and ΔMAE) of 0.02, 0.59, and 0.94, respectively. In contrast, the XGB-based model showed evidence of overfitting, as indicated by ΔRMSE of 7.22 and ΔMAE of 2.80.

In contrast, the XGB-based model showed slightly higher discrepancies between the training and validation results (ΔRMSE = 7.22 and ΔMAE = 2.80), indicating a moderate tendency toward overfitting due to its higher model complexity. However, the ΔR^2^ value (0.20) remained within an acceptable range, and the model still demonstrated reasonable generalization performance after hyperparameter tuning and cross-validation. These differences were primarily attributed to the nonlinear and ensemble characteristics of the XGBoost algorithm, which can produce higher variance when dealing with limited or heterogeneous samples.

**Figure 8:**
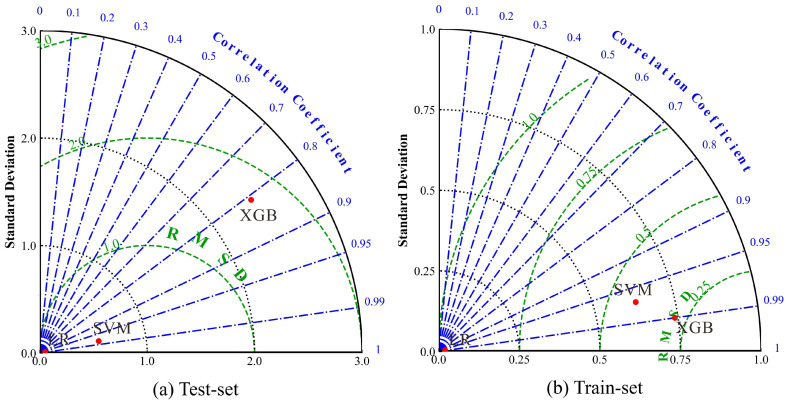
Taylor diagrams of different machine learning methods for: (**a**) Test set; (**b**) Training set.

The Taylor diagram provides a comprehensive assessment of model performance by simultaneously representing three key statistical parameters: correlation, variability, and error [65,66]. As illustrated in Figure 8, the radial distance from the origin denoted the standard deviation, the azimuthal angle reflected the correlation coefficient between model predictions and the reference data, and the concentric contour lines indicated the centered RMSD. Thus, in this diagram, model points that were located closer to the reference point on the *x*-axis suggested higher overall agreement with the observations. And this reflected both stronger correlation and smaller deviations. The results clearly indicated that LR demonstrated superior performance compared with the other two models.

**Figure 9:**
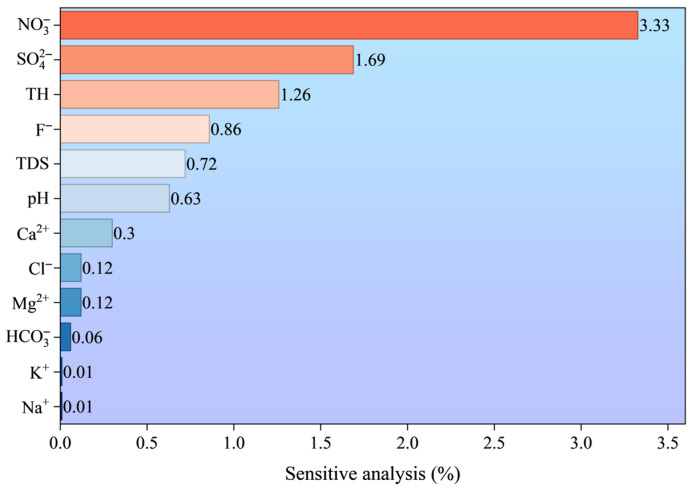
Sensitivity analysis of the LR prediction model.

To further evaluate the influence of each input parameter on the LR model, a sensitivity analysis was conducted. The results (Figure 9) indicated that NO_3_^−^ was the most important input variable, with its variation exerting the greatest impact on the WQI prediction results. SO_4_^2−^ and TH were the next most influential parameters, ranking second and third, respectively.

## 5. Conclusions

This study comprehensively employed multiple approaches to investigate the hydrochemical characteristics and formation mechanisms of groundwater in the study area, and separately evaluated and predicted the drinking water quality based on game theory and machine learning methods. The main conclusions are as follows:

(1) The groundwater in the study area is weakly alkaline fresh water, with hardness ranging from soft to medium. The anions and cations with the highest contents are HCO_3_^−^ and Ca^2+^, respectively, and the hydrochemical type is dominated by the Ca-HCO_3_ type.
(2) Water-rock interaction is the dominant factor influencing the hydrochemical characteristics of groundwater. The formation of hydrochemistry is jointly affected by the weathering and dissolution of carbonate rocks and silicate rocks, accompanied by cation exchange and adsorption. In addition, groundwater is also subject to disturbances from certain human activities, such as agricultural practices and landfill disposal.
(3) More than 95% of groundwater samples are considered as excellent water quality, suggesting that the groundwater quality is generally good and suitable for drinking in the study area. The extremely poor water quality may indicate local pollution.
(4) Among the three machine learning models (LR, SVM, XGB) constructed for groundwater quality prediction, the LR model exhibits the optimal performance, with the highest prediction accuracy and strongest stability, followed by the SVM model. The XGB model, however, shows obvious overfitting and lower generalization ability. The LR model can provide reliable technical support for the dynamic monitoring and early warning of groundwater quality in the study area. The results of the sensitivity analysis indicated that NO_3_^−^ was the most influential variable affecting the performance of the LR model.

Since the current sampling is based on pre-existing monitoring wells, the spatial distribution of sampling points is inherently uneven, leading to a higher number of samples collected in Rikaze and Benbu compared to Bailang. In addition, this study does not sufficiently account for temporal changes in groundwater quality, which can vary seasonally. To address these issues, sampling scope will be expanded by incorporating domestic water sources used by local residents, thereby improving the representativeness and overall quality of the dataset in future studies; the temporal changes in groundwater quality can be predicted combining machine learning and other methods, or continuously collect time-series monitoring data to more accurately analyze the seasonal variation in groundwater quality.

In addition, the three machine learning models (LR, SVM, and XGB) exhibited strong predictive performance and stability through 5-fold cross-validation; however, some limitations should still be acknowledged. Due to the lack of independent datasets, external validation could not be conducted in this study. Instead, 5-fold cross-validation was adopted to ensure model robustness and mitigate overfitting, which effectively enhanced the reliability of the results. Furthermore, the current analysis was based on groundwater samples collected primarily during a single hydrological season (dry period). As a result, temporal variations in groundwater quality, which may occur seasonally due to changes in recharge, temperature, and anthropogenic activities, were not fully captured. Future research should incorporate multi-seasonal or long-term monitoring data to better characterize the temporal dynamics of groundwater chemistry and their influence on model performance. The models demonstrated excellent predictive performance within the study area, but they cannot be directly applied to other regions worldwide without proper recalibration and adaptation. Differences in hydrogeological, climatic, and anthropogenic conditions may significantly affect groundwater quality patterns and model applicability. Therefore, future research should aim to integrate broader-scale and multi-source datasets, improve model transferability across diverse hydrogeological settings, and develop hybrid frameworks that combine data-driven and process-based approaches to enhance the global applicability and reliability of ground-water quality prediction.

## Figures and Tables

**Table 1:** The reference value of RI.

*n*	1	2	3	4	5	6	7	8	9	10	11	12	13	14	15
*RI*	0	0	0.52	0.89	1.12	1.26	1.36	1.41	1.46	1.49	1.52	1.54	1.56	1.58	1.59

**Table 2:** Statistical characteristics of hydrochemical parameters of groundwater samples (mg/L).

Parameters	pH	TDS	TH	K^+^	Na^+^	Ca^2+^	Mg^2+^	Cl^−^	SO_4_^2−^	HCO_3_^−^	NO_3_^−^	F^−^
Min	7.00	69.00	15.00	0.15	2.24	5.63	0.20	1.01	1.45	39.90	0.02	0.03
Median	8.00	226.00	173.00	1.08	12.21	52.13	9.46	2.87	50.49	165.90	0.98	0.13
Mean	8.08	302.58	218.91	1.52	17.08	60.19	15.68	10.46	82.29	170.43	2.95	0.16
Max	9.10	1660.00	1370.00	20.30	172.90	330.75	144.13	443.70	951.66	519.75	73.50	0.79
CV	0.05	0.77	0.82	1.45	1.12	0.68	1.26	4.12	1.42	0.42	2.97	0.70
Limit	6.5–8.5	450	1000	200	200	200	150	250	250	450	20	1
Exceedance	12.90%	16.13%	0.81%	0.00%	0.00%	0.81%	0.00%	0.81%	0.81%	1.61%	4.03%	0.00%

**Table 3:** The optimal parameters used in the Machine-learning model.

Models	LR	SVM	XGB
Hyperparameters	Default	kernel = ‘rbf’ C = 100 gamma = 0.01 epsilon = 0.1	n_estimators = 150 max_depth = 3 learning_rate = 0.05 subsample = 0.8 colsample_bytree = 1

**Table 4:** Model evaluation values of the training set and test set.

Parameters	Model	R^2^	RMSE	MAE	ΔR^2^	ΔRMSE	ΔMAE
Test set	LR	0.99	0.00	0.00	-	-	-
	SVM	0.98	1.55	0.56	-	-	-
	XGB	0.81	6.41	2.43	-	-	-
Training set	LR	0.99	0.00	0.00	0.00	0.00	0.00
	SVM	0.97	4.01	0.63	0.02	0.59	0.94
	XGB	0.99	1.22	0.74	0.20	7.22	2.80

Note: Δ indicates the deviation between the metrics calculated on the training dataset and those from 5-fold cross-validation.

## Data Availability

The original contributions presented in this study are included in the article/Supplementary Material. Further inquiries can be directed to the corresponding author.

## Supplementary Materials

The following supporting information can be downloaded at: https://www.mdpi.com/article/10.3390/toxics13110985/s1, Text S1: Predicted models of water quality [67,68,69,70]; Text S2: One-at-a-Time (OAT)-Based Sensitivity Analysis; Table S1: The judgment matrix criterion layer; Table S2: The judgment matrix solution layer 1; Table S3: The judgment matrix solution layer 2.
