# Supplementary material for: Improved Assessment and Prediction of Groundwater Drinking Quality Integrating Game Theory and Machine Learning in the Nyangchu River Basin, Southwestern Qinghai–Tibet Plateau
Source: Toxics. 2025 Nov 16;13(11):985. doi: 10.3390/toxics13110985 (PMC12656682; doi:10.3390/toxics13110985)
Supplement: Supplementary file 1 [file toxics-13-00985-s001.zip › toxics-3940001-supplementary.pdf]

Supplementary Materials

1. Predicted models of water quality

(1) Linear regression (LR)

Linear regression (LR) is a statistical learning method employed to investigate the relationship between one or more independent variables and a dependent variable. The fundamental assumption of the model is that the target value can be represented as a linear combination of the input features with an additional error term. The regression coefficients are estimated using the method of least squares, which determines the optimal straight line or high-dimensional hyperplane that best fits the data by minimizing the sum of squared residuals [1,2]. This fitted model captures the underlying data trend and enables prediction for new observations. The fundamental principle of the formula is presented in Eq. (1).

$$y_i=\beta_0+\beta_1 x_{i1}+\beta_2 x_{i2}+\cdots+\beta_p x_{ip}+\varepsilon_i, \quad i=1,2,\ldots,n \tag{1}$$

where β_0 is the intercept, β_p is the regression coefficients, and ε_i is the error term. The coefficients β is estimated using ordinary least squares (OLS) to minimize the sum of squared residuals.

(2) Support vector machine (SVM)

Support Vector Machine (SVM) is a supervised learning method. Its core principle is to identify an optimal separating hyperplane in the feature space that maximally separates samples of different classes while maximizing the margin between them. The position of this hyperplane is determined solely by a subset of critical samples, known as support vectors, which enhances the model's generalization capability [1]. For nonlinearly separable data, SVM maps the input features into a higher-dimensional space using a kernel function. The decision function is expressed as Eq. (2).

$$f(x)=sign\left(\sum_{i\square SV}\alpha_i y_i K(x_i,x)+b\right) \tag{2}$$

where α_i is the Lagrange multipliers corresponding to the support vectors; y_i is the class label of the i-th support vector; b is the bias term; $K(x_i, x)$ is the kernel function, which implicitly performs the mapping to a higher-dimensional feature space.

(3) Extreme Gradient Boosting (XGB)

Extreme Gradient Boosting (XGB) is a library that implements Gradient Boosting Decision Trees (GBDT) and provides an efficient improvement over the standard GBDT algorithm [3]. Its core design philosophy focuses on enhancing the procedure for constructing single CART regression trees within the GBDT framework. The construction of a CART regression tree generally involves two key steps: (1) partitioning the input dataset through multiple splits to generate a set of optimal regions or leaf nodes RRR; and (2) calculating the output value ccc for each region or leaf node. In XGBoost, these two steps are addressed jointly, such that the partitioning of regions and the determination of their optimal output values are solved simultaneously [4]. This joint optimization directly minimizes the GBDT loss function, and the process can be formally expressed as the following objective function (Eq. (3)).

$$L^{(t)}=-\frac{1}{2}\sum_{j=1}^{T}\frac{\left(\sum_{i\in I_j} g_i\right)^2}{\sum_{i\in I_j} h_i+\lambda}+\gamma T \tag{3}$$

where T is the number of leaf nodes; I_j denotes the instance set of the j-th leaf; g_i and h_i are the first- and second-order gradients of the loss function; γ and λ are regularization parameters.

2. One-at-a-Time (OAT)-Based Sensitivity Analysis

To evaluate the relative influence of each input feature on the model output, a One-at-a-Time (OAT) sensitivity analysis was performed. This method involves perturbing one input variable at a time while keeping all other variables fixed at their baseline values, thereby quantifying the direct impact of that variable on model predictions.

In this study, the first sample from the testing dataset was selected as the baseline condition. For each input feature X_i, its value was increased and decreased by 10% (+10% and −10%) relative to the baseline. The model was then used to predict the corresponding outputs $Y_i^{+10\%}$ and $Y_i^{-10\%}$. The base prediction Y_0 was also obtained under the original conditions. The sensitivity index for each feature was computed as Eq. (4)

$$S_i=\frac{|Y_i^{+10\%}-Y_0|+|Y_i^{-10\%}-Y_0|}{2\times Y_0}\times 100\% \tag{4}$$

where, S_i represents the percentage change in the model output caused by a ±10% variation in the corresponding input feature. A larger S_i indicates a higher sensitivity of the model output to that input variable. All features were ranked according to their sensitivity indices to determine the

dominant controlling factors in the prediction process.

Table S1 The judgment matrix criterion layer.

	Low hazard index	Medium hazard index
Low hazard index	1	1/3
Medium hazard index	3	1

Table S2 The judgment matrix solution layer 1.

	pH	TDS	TH	K	Na	Ca	Mg	Cl	SO_4	HCO_3
pH	1	3	3	5	5	5	5	5	5	5
TDS	1/3	1	1	3	3	3	3	3	3	3
TH	1/3	1	1	3	3	3	3	3	3	3
K	1/5	1/3	1/3	1	1	1	1	1/5	1/3	1/3
Na	1/5	1/3	1/3	1	1	1	1	1/5	1/3	1/3
Ca	1/5	1/3	1/3	1	1	1	1	1/5	1/3	1/3
Mg	1/5	1/3	1/3	1	1	1	1	1/5	1/3	1/3
Cl	1/5	1/3	1/3	5	5	5	5	1	1/3	1/3
SO_4	1/5	1/3	1/3	3	3	3	3	3	1	1/3
HCO_3	1/5	1/3	1/3	3	3	3	3	3	3	1

Table S3 The judgment matrix solution layer 2.

	NO_3	F
NO_3	1	1/3
F	3	1

Reference

1. Nafouanti, M.B., Li, J., Mustapha, N.A., Uwamungu, P., AL-Alimi, D., 2021. Prediction on the fluoride contamination in groundwater at the datong basin, northern China: Comparison of random forest, logistic regression and artificial neural network. Applied Geochemistry 132, 105054. https://doi.org/10.1016/j.apgeochem.2021.105054
2. Tsuchihara, T., Yoshimoto, S., Shirahata, K., Nakazato, H., Ishida, S., 2023. Analysis of groundwater-level fluctuation and linear regression modeling for prediction of initial groundwater level during irrigation of rice paddies in the nasunogahara alluvial fan, central japan. Environ Earth Sci 82, 473. https://doi.org/10.1007/s12665-023-11174-w.
3. Gholami, V., Khaleghi, M.R., Salimi, E.T., 2025. Comparison of extreme gradient boosting, deep learning, and self-organizing map methods in predicting groundwater depth. Environ

Earth Sci 84, 179. https://doi.org/10.1007/s12665-025-12183-7

4. Zhang, K., Wang, X., Liu, T., Wei, W., Zhang, F., Huang, M., Liu, H., 2024. Enhancing water quality prediction with advanced machine learning techniques: An extreme gradient boosting model based on long short-term memory and autoencoder. Journal of Hydrology 644, 132115. https://doi.org/10.1016/j.jhydrol.2024.132115
